# Interaction between intratumoral microbiota and tumor mediates the response of neoadjuvant therapy for rectal cancer

**DOI:** 10.3389/fmicb.2023.1229888

**Published:** 2023-10-12

**Authors:** Lejia Sun, Jiangming Qu, Xindi Ke, Yue Zhang, Hengyi Xu, Ning Lv, Jingze Leng, Yanbin Zhang, Ai Guan, Yifei Feng, Yueming Sun

**Affiliations:** ^1^Department of General Surgery, The First Affiliated Hospital of Nanjing Medical University, Nanjing, China; ^2^The First School of Clinical Medicine, Nanjing Medical University, Nanjing, China; ^3^Colorectal Institute of Nanjing Medical University, Nanjing, China; ^4^Chinese Academy of Medical Sciences & Peking Union Medical College, Beijing, China; ^5^School of Medicine, Tsinghua University, Beijing, China; ^6^Department of Spine Surgery, Beijing Jishuitan Hospital, Beijing, China

**Keywords:** locally advanced rectal cancer, neoadjuvant chemoradiotherapy, intratumoral microbiota, cancer-associated fibroblasts, biomarker

## Abstract

**Background:**

Previous observations have demonstrated that the response to neoadjuvant chemoradiotherapy (nCRT) is highly variable in patients with locally advanced rectal cancer (LARC). Recent studies focusing on the intratumoral microbiota of colorectal cancer have revealed its role in oncogenesis and tumor progression. However, limited research has focused on the influence of intratumoral microbiota on the nCRT of LARC.

**Methods:**

We explored the microbial profiles in the tumor microenvironment of LARC using RNA-seq data from a published European cohort. Microbial signatures were characterized in pathological complete response (pCR) and non-pCR groups. Multi-omics analysis was performed between intratumor microbiomes and transcriptomes.

**Results:**

Microbial α and β diversity were significantly different in pCR and non-pCR groups. Twelve differential microbes were discovered between the pCR and non-pCR groups, six of which were related to subclusters of cancer-associated fibroblasts (CAFs) associated with extracellular matrix formation. A microbial risk score based on the relative abundance of seven differential microbes had predictive value for the nCRT response (AUC = 0.820, *p* < 0.001).

**Conclusion:**

Our study presents intratumoral microbes as potential independent predictive markers for the response of nCRT to LARC and demonstrates the underlying mechanism by which the interaction between intratumoral microbes and CAFs mediates the response to nCRT.

## Introduction

Colorectal cancer (CRC) is the third most commonly diagnosed cancer in males and second in females (Bray et al., 2018). Approximately two-thirds of CRC arise in the rectum. At least 25% of cases are at an advanced stage at initial diagnosis (Aklilu and Eng, 2011; Glynne-Jones et al., 2017). Neoadjuvant chemoradiotherapy (nCRT) has been established as a standard treatment for patients with locally advanced rectal cancer (LARC) to achieve higher sphincter preservation rates and lower locoregional recurrence rates (Sauer et al., 2004). Good responses to nCRT improve local control and can shift clinical strategies from radical surgery to “watch and wait.” However, observations from many clinical studies have demonstrated that the response to nCRT is highly variable in LARC (Loos et al., 2013; Petrelli et al., 2020). Approximately 20–30% of patients with rectal cancer achieve a complete response to conventional nCRT (Cercek et al., 2018; Buckley et al., 2020; van der Sluis et al., 2020). Since nCRT could also cause treatment-associated toxicities (Pucciarelli et al., 2011; West et al., 2014), and a good response to nCRT may lead to “watch and wait” instead of surgery, it is essential to determine whether patients can potentially benefit from nCRT based on pre-treatment biomarkers.

Recent studies have detected the presence of microbiota in tumor tissues (Nejman et al., 2020; Poore et al., 2020). Most cancer microbes are present in cells. The cancer microbiota can influence biological behavior by modulating tumorigenesis and chemotherapy resistance. Previous studies focusing on the intratumoral microbes of CRC have revealed the role of the intratumoral microbiota in oncogenesis and tumor progression (Dejea et al., 2018; Rubinstein et al., 2019; Liu et al., 2021). Intratumoral bacteria can affect the therapeutic response by metabolizing drugs into inactive forms and influencing antitumor immunity (Da Cunha et al., 2022; Wang et al., 2022). Therefore, it is rational to speculate that the intratumoral microbiome may affect nCRT responses. Recent studies have investigated the predictive value of the gut microbiome in response to nCRT in patients with LARC (Jang et al., 2020; Shi et al., 2020; Yi et al., 2021). However, Zhang et al. (2021) demonstrated different patterns of intratumoral microbial profiles from either the gut microbiome or the mucosal microbiome. To our knowledge, no research has focused on the influence of intratumoral microbial profiles on chemoradiation therapy.

In this study, we analyzed the intratumoral microbiota of rectal cancer and the correlation between microbes and the tumor microenvironment (TME) based on RNA-seq data of a large LARC cohort. We profiled the microbiome composition in LARC according to nCRT response and demonstrated significantly different microbial signatures in the pathologically complete response (pCR) and non-pCR groups. We also established an intratumoral microbiome signature to identify patients likely to benefit from preoperative nCRT for clinical application. Interactions between intratumoral microbes and cancer-associated fibroblasts were also detected, revealing the potential mechanisms underlying the relationship between microbiota and therapeutic responses.

## Materials and methods

### Patients and datasets

The pre-treatment raw RNA sequences, gene expression data, and clinicopathologic data of a published cohort (*n* = 105 rectal cancer patients) were obtained from the Gene Expression Omnibus (GEO) repository (GSE 190826) (Nicolas et al., 2022). In this cohort, patients with histologically confirmed rectal carcinoma with an inferior margin of no more than 12 cm above the anal verge with perirectal fat infiltration (cT3–4) or lymph node involvement (cN+) were treated with standard nCRT followed by surgical resection (Lee et al., 2020).

### Transcriptomic analysis

Differential gene expression analysis was conducted using the R package limma, with significant differences required to fulfill the criteria |log2 Fold Change| > 1 and false discovery rate (FDR) < 0.05. cancer-associated fibroblasts (CAFs) derived from a single-cell analysis of rectal cancer tissue (GSE132465) were clustered into 18 subpopulations using the Leiden algorithm (Traag et al., 2019). CIBERSORTx calculated and estimated the immune infiltration of 22 types of common immune cells and 18 subpopulations of CAFs (Newman et al., 2019). Spearman’s correlation coefficient was calculated to measure the connections between the microbial and cellular abundance of immune cells and CAFs. Weighted gene co-expression network analysis (WGCNA) was used to generate a co-occurrence network based on the relative abundances of each core genera performed by ImageGP with default parameters (Chen et al., 2022). Pathway and process enrichment analysis of hub genes derived from WGCNA and the marker genes of each subpopulation of CAFs were performed using the Metascape tool (Han et al., 2018). The protein–protein interaction network of the signature genes of each subpopulation of CAFs was constructed based on protein–protein physical interactions according to the database of BioGrid and STRING (physical score > 0.132) performed by Metascape. The Molecular Complex Detection (MCODE) algorithm (Bader and Hogue, 2003) has been applied to identify densely connected network components and the enrichment analysis of transcription factors using the transcriptional regulatory network (TRRUST) module of Metascape.

### Microbial data processing and analysis

Raw RNA sequencing data from preoperative endoscopic biopsies underwent quality control with FastQC and then were trimmed to remove adapter sequences using the Cutadapt tool (version 4.1). Subsequently, the reads underwent taxonomic identification and were assigned to humans, bacteria, archaea, viruses, protozoa, and fungi by Kracken2 based on the Standard Kraken2 reference database plus protozoa and fungi (Wood et al., 2019). Reestimation of reads was performed by Bayesian Reestimation of Abundance with Kraken (Bracken) to compute a highly accurate genus-level abundance (Lu et al., 2017). Only the sequences assigned to bacteria were preserved, with sequences assigned to humans, archaea, viruses, and fungi removed. Raw abundance data for bacteria were decontaminated using the decontam algorithm and rarefied (Davis et al., 2018).

Α diversity was calculated using the R package “vegan” and was displayed as the Shannon index. β-diversity was analyzed using principal coordinate analysis (PCoA) based on Jaccard’s distance and partial least squares discrimination analysis (PLS-DA). Linear discriminant analysis effect size (LEfSe) was used to evaluate differential taxa between the pCR and non-pCR groups (Segata et al., 2011). Differences in relative abundance at the genus level were also analyzed and visualized using STAMP software (Welch’s t-test with Benjamini–Hochberg FDR < 0.05) (Parks et al., 2014). Microbial network analysis was conducted using the R package “ggClusterNet” (Wen et al., 2022). The microbial and CAF network was generated by Spearman’s correlation with a threshold of correlation coefficient > 0.3 and *p* < 0.05. Receiver operating characteristic (ROC) curves were plotted, and the area under ROC curves (AUC) was calculated to evaluate the ability of intratumoral microbes to predict the therapeutic effect of nCRT, which was also analyzed using logistic regression analysis.

## Results

### Population characteristics

The clinical characteristics of the patients are summarized in Table 1. A total of 105 patients who met the inclusion/exclusion criteria were included in the study. Among them, 68.8% were male and 31.2% were female. The median age of the cohort was 65 (range 34–86). LARC was graded as grade 0 in two patients (1.9%), grade 2 in 95 patients (92.2%), and grade 3 in six patients (5.8%) by biopsy. The number of patients with cTNM stages I, II, III, and IV was 2 (2.0%), 12 (11.8%), 87 (85.3%), and 1 (1.0%), respectively. Seventy-six patients (72.4%) received treatment of 50.4 Gy + 5-FU, 26 patients (24.8%) received 50.4 Gy + 5-FU/Ox, and three patients (2.8%) received 50.4 Gy + Capecitabine. After subsequent surgery, 26 patients (24.8%) achieved pCR, and 79 (75.2%) did not. The median disease-free survival (DFS) of the cohort was 50 (range 2–132) months.

**Table 1:** Clinical characteristics of patients with LARC.

Patients	*n* = 105
Sex	
Male Female	64 (68.8%) 41 (31.2%)
Age (years)	65 (34–86)
Grading	*n* = 103
0	2 (1.9%)
2	95 (92.2%)
3	6 (5.8%)
cTNM	*n* = 102
I	2 (2.0%)
II	12 (11.8%)
III	87 (85.3%)
IV	1 (1.0%)
Treatment	
50.4 Gy + 5-FU	76 (72.4%)
50.4 Gy + 5-FU/Ox	26 (24.8%)
50.4 Gy + Capecetabine	3 (2.8%)
Therapeutic response	
non-pCR	79 (75.2%)
pCR	26 (24.8%)
DFS (months)	50 (2–132)

### Different microbial profiles in patients with distinct therapeutic responses

**Figure 1:**
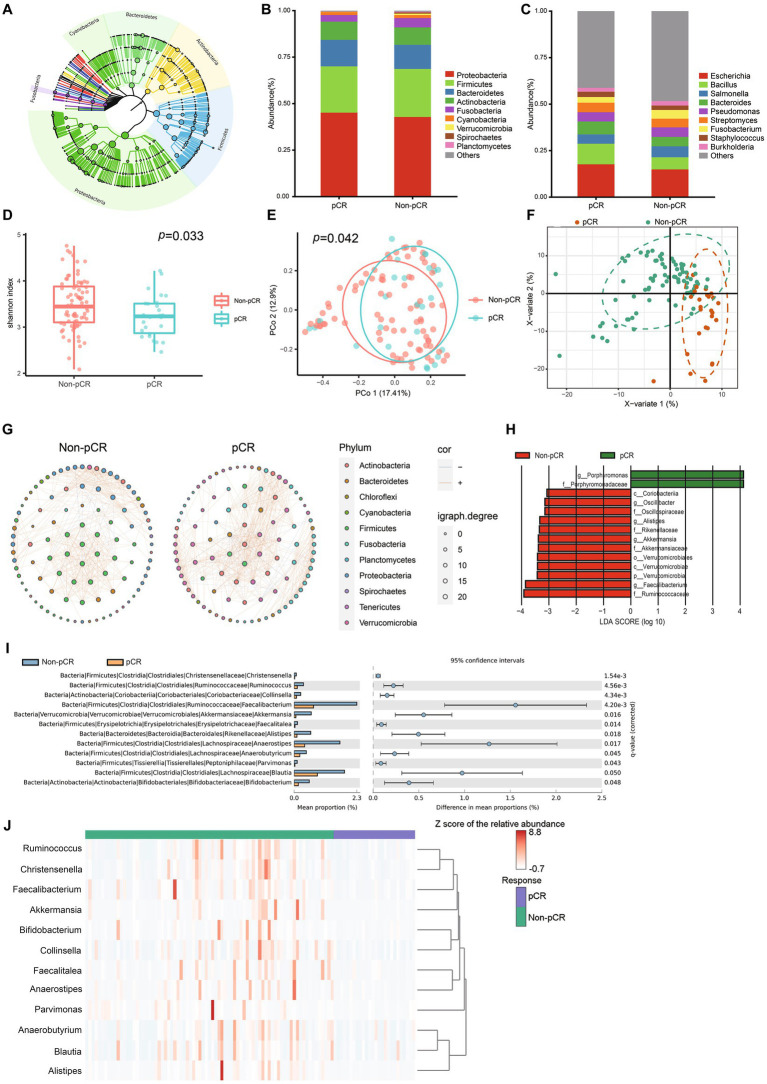
Characterization of the microbiome in the pCR and non-pCR groups. **(A)** Phylogenetic tree of the intratumoral microbiome in rectal adenocarcinoma. Each node represents a specific taxon. Different colors denote different phyla. The size of the nodes is related to the abundance of each taxon. **(B)** Stack plots of microbial composition at the phylum level. **(C)** Stack plots of microbial composition at the genus level. **(D)** The α diversity of samples belonging to the pCR and non-pCR groups. **(E)** Principal coordinates analysis (PCoA) based on the Jaccard distance of the pCR and non-pCR groups. **(F)** Partial least squares discriminant analysis (PLS-DA) of the pCR and non-pCR groups. **(G)** Microbial networks in the pCR and non-pCR groups. Each dot represents one genus. Only 150 genera with top abundance are shown. The size of each dot represents the igraph degree, which reflects the number of significant correlations between the corresponding genus with the others. The orange and blue lines indicate positive and negative correlations, respectively. **(H)** Histogram of linear discriminant analysis (LDA) scores computed from features differentially abundant between the pCR and non-pCR groups. Features with LDA score (log10) > 3.0 and *p* < 0.05 are shown. **(I)** Bar plot of relative abundance of genera with significant differences between pCR and non-pCR groups. Comparison is conducted *via* Welch’s *t*-test, and only genera with Benjamini–Hochberg false discovery rate < 0.05 are shown. **(J)** Heatmap indicates the correlation between the therapeutic response and microbial profiles.

After taxonomic classification and the removal of human reads, we assessed the landscape of the intratumoral microbiome abundance profiles in all available pre-treatment samples in patients with rectal cancer. Figure 1A shows the evolutionary structure of the microbial profiles in the cohort. Proteobacteria, Firmicutes, Bacteroidetes, Actinobacteria, and Fusobacteria were the top five abundant microbes at the phylum level (Figure 1B). Further analysis at the genus level also revealed the most abundant microbes (Figure 1C). There were 18 genera and 103 genera specific to the pCR group and the non-pCR group, respectively, with 293 genera shared by both groups (Supplementary Figure S1A). The top 10 abundant genera along with their taxonomic information at different levels, were summarized in Supplementary Figure S1B.

We observed higher α diversity in the non-pCR group than in the pCR group (Shannon index, *p* = 0.033) (Figure 1D). PCoA analysis by Jaccard’s distance also revealed significant differences in β diversity between the two groups (*p* = 0.042) (Figure 1E). PLS-DA also indicated significant differences between the microbial communities in pCR and non-pCR (Figure 1F). We also observed different network patterns in the intratumoral microbes of the pCR and non-pCR groups (Figure 1G). Multiple correlations between Firmicutes and Proteobacteria were observed in the non-pCR group, which differed from the pCR group.

LEfSe analysis was conducted to assess the differences in microbial signatures between the pCR and non-pCR groups. Fourteen differential taxa were observed between pCR and non-pCR groups, including genera *Faecalibacterium, Alistipes,* and *Akkermansia,* etc. (Figure 1H). Further analysis using the STAMP software presented the presence of differential microbes at the genus level (Figure 1I). We observed that the abundances of *Christensenella, Ruminococcus, Collinsella, Faecalibacterium, Akkermansia, Faecalitalea, Alistipes, Anaerostipes, Anaerobutyricum, Pavimonas, Blautia*, and *Bifidobacterium* were significantly enriched in the non-pCR group. When summarizing the abundance of these 12 microbes, we observed distinct microbial patterns between the non-pCR and pCR groups, as shown in the heatmap (Figure 1J).

### Interaction between intratumoral microbes and tumor

**Figure 2:**
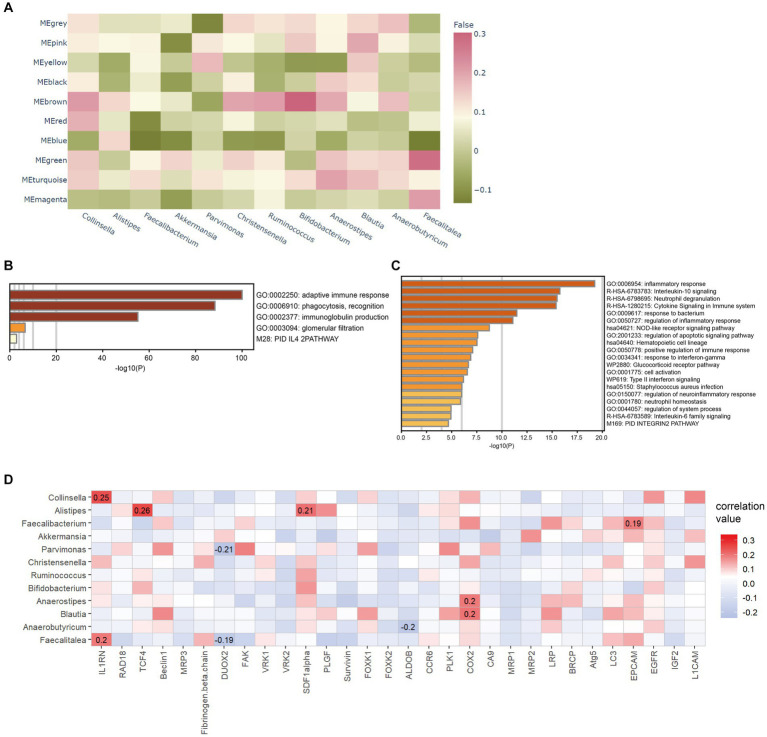
Correlation between intratumoral microbes and tumor microenvironment. **(A)** Heatmap indicates the correlation between identified modules and differential microbes. **(B)** Bar graph of enriched pathways of the brown module in **(A)**. **(C)** Bar graph of enriched pathways of the green module in **(A)**. **(D)** Correlations between the expression profile of treatment-resistant gene and differential microbes. The correlation coefficient is displayed if *p* < 0.05.

We performed further multi-omics analysis, combining microbiota, gene expression, and immune and stromal cell infiltration, to better understand the interaction between intratumoral microbes and the tumor. Based on WGCNA, *Faecalitalea* and *Clollinsella* were significantly correlated with co-expression modules associated with adaptive immune response, phagocytosis, recognition, and inflammatory response (Figures 2A–C). Correlation analysis revealed several significant but not robust associations between intratumoral bacteria and previously reported resistant gene expression (Figure 2D; Li et al., 2022). It also indicated a significant association between specific intratumoral microbiomes and eosinophils, neutrophils, macrophages M2 and naïve B cell infiltration (Supplementary Figure S2). These findings indicated that interactions may occur between the intratumoral microbiome and TME through multifaceted mechanisms.

**Figure 3:**
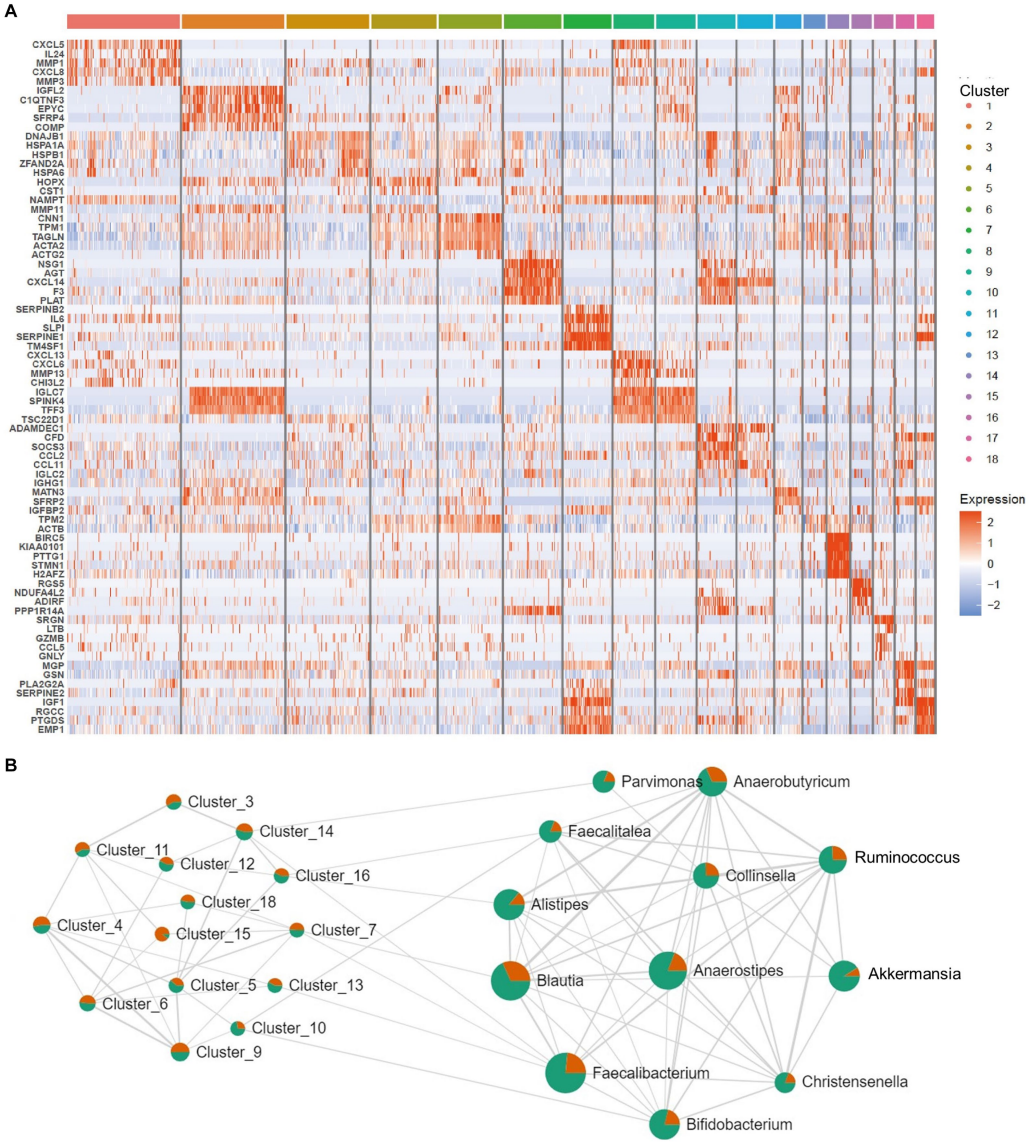
Correlation between intratumoral microbes and cancer-associated fibroblasts (CAFs). **(A)** Heatmap for marker genes of 18 CAF clusters in the tumor microenvironment (TME). **(B)** Network patterns between the 18 clusters of CAFs and the 12 differential microbes. The strings represent a correlation coefficient > 0.3 with *p* < 0.05.

**Figure 4:**
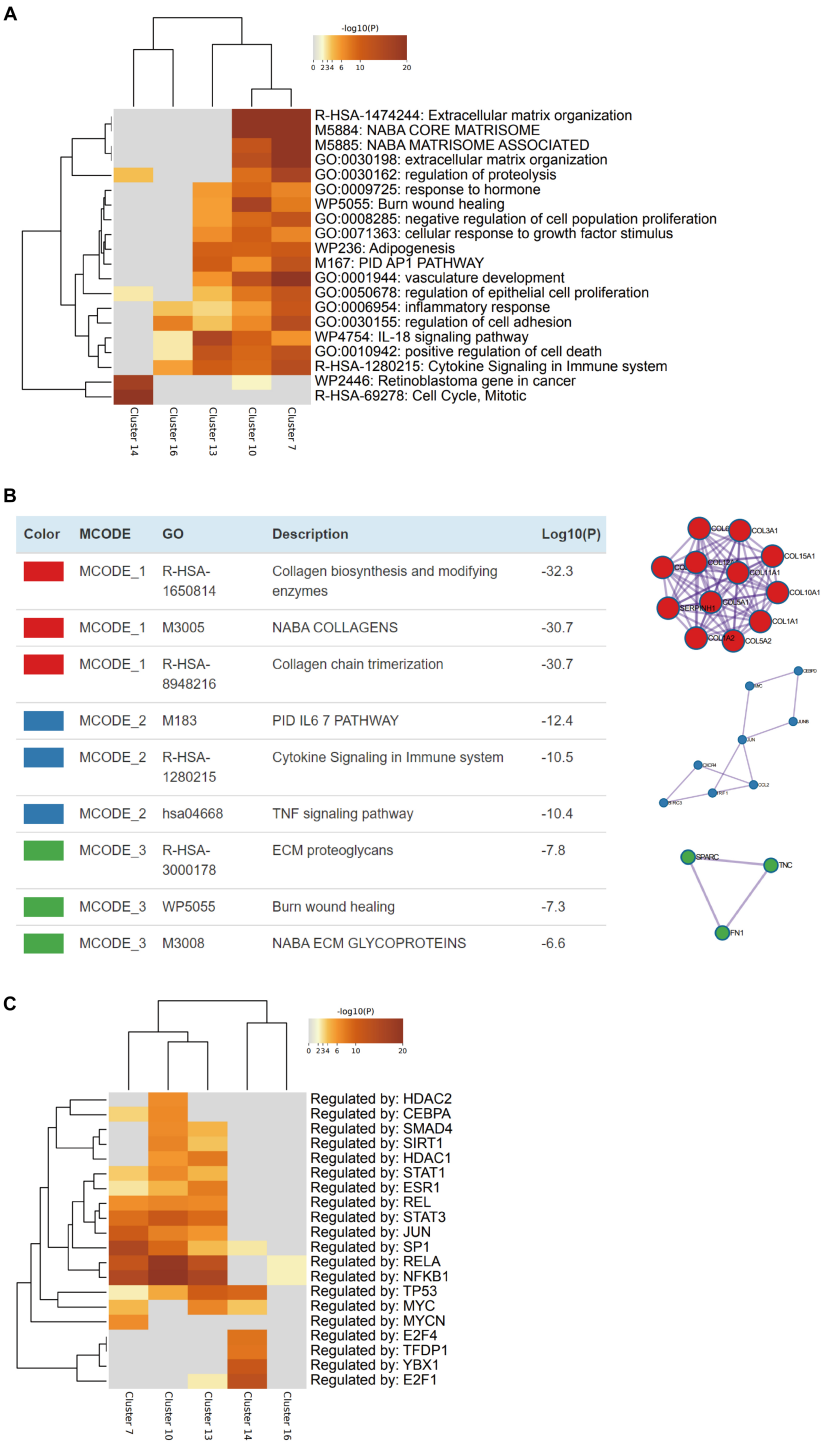
Characterization of the cancer-associated fibroblasts (CAFs) that are related to intratumoral microbiomes. **(A)** Heatmap indicates the pathways/processes enriched in each cluster of CAFs. **(B)** Protein–protein physical interactions and GO enrichment analysis of MCODE network components of the marker genes of CAF cluster 10. **(C)** Heatmap indicates the upstream regulatory factors for each cluster of CAFs.

After the CAFs of rectal cancer were clustered into 18 subpopulations (Figure 3A), we assessed the relationship between the abundance of 12 characteristic microbes and the infiltrating CAF clusters within our cohort. We observed clusters 7, 10, 13, 14, and 16 correlated with six genera in the network (Figure 3B). Enrichment Analysis demonstrated that CAF clusters 7 and 10 were associated with collagen biosynthesis, modification, and ECM organization and modulation (Figures 4A,B and Supplementary Figure S3). Moreover, the presumed upstream regulatory factors of both clusters 7 and 10 include NF-κB (Figure 4C).

### Establishment of a predictive classifier for response to nCRT

**Figure 5:**
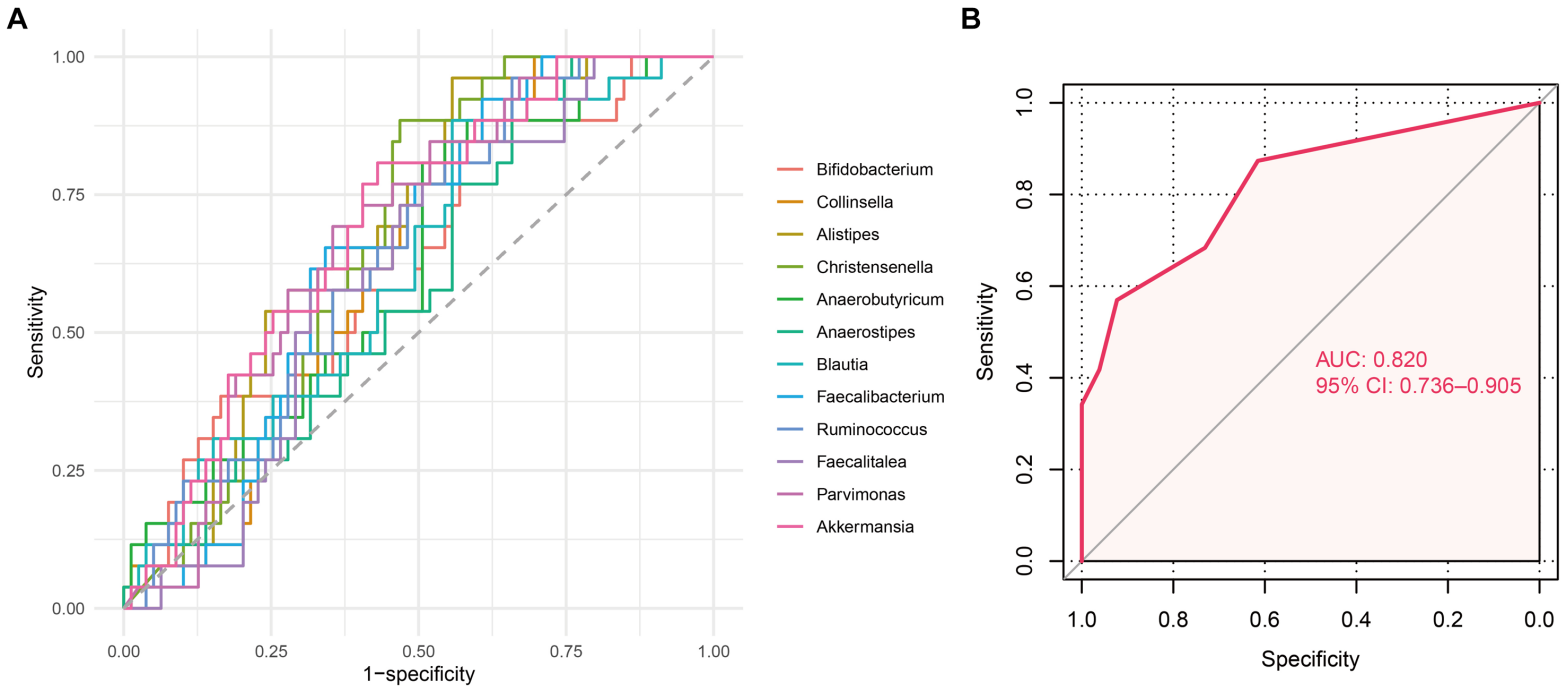
Predictive value of differential microbes for nCRT response. **(A)** Receiver operator characteristic (ROC) analysis of 12 differential microbes between the pCR and non-pCR groups predicting the nCRT response. **(B)** ROC analysis of the microbial risk score predicting the nCRT response.

To translate our findings into clinical applications, we established a predictive classifier for response to nCRT. Thus, we conducted ROC analysis and calculated the area under ROC curves (AUC) of the 12 differential microbes, among which, *Collinsella* (AUC = 0.629, *p* = 0.049), *Alistipes* (AUC = 0.685, *p* = 0.005), *Christensenella* (AUC = 0.672, *p* = 0.009), Faecalibacterium (AUC = 0.647, *p* = 0.025), *Ruminococcus* (AUC = 0.645, *p* = 0.027), *Pavimonas* (AUC = 0.682, *p* = 0.005), and *Akkermansia* (AUC = 0.693, *p* = 0.003) appeared significantly predictive of the therapeutic response (Figure 5A and Supplementary Table S1). Then, patients were classified into the high-level group or low-level group according to the relative abundance of these seven microbes. The optimal cutoff value for each of these seven microbes was set at the maximum Youden index. After the patients being stratified by the corresponding cutoff values, *Collinsella* (AUC = 0.652, *p* = 0.021), *Alistipes* (AUC = 0.702, *p* = 0.002), *Christensenella* (AUC = 0.702, p = 0.002), *Faecalibacterium* (AUC = 0.658, *p* = 0.016), *Ruminococcus* (AUC = 0.652, *p* = 0.021), *Pavimonas* (AUC = 0.669, *p* = 0.010), and *Akkermansia* (AUC = 0.689, *p* = 0.004) were still significantly associated with the responses to nCRT (Supplementary Figure S4 and Supplementary Table S2). We assigned a score of 0 to the low-level group and a score of 1 to the high-level group. Then, the microbial risk score of each individual was defined by the sum value of the scores based on these seven predictive microbes. The microbial risk score was significantly linked to the therapeutic outcomes, with the AUC of the microbial risk score for predicting non-pCR being 0.820 (*p* < 0.001) (Figure 5B). As shown in Table 2, multiple logistic regression analysis revealed that the microbial risk score was an independent predictive factor for therapeutic response to nCRT (HR = 2.253, 95% CI: 1.483–3.422, *p* < 0.001). For each one score increase in the microbial risk score, the risk of non-pCR was more than doubled.

**Table 2:** Multiple logistic regression of clinical characteristics for predicting responses to neoadjuvant chemoradiotherapy.

Risk factors	OR (95% CI)	Value of *p*
Gender	0.777 (0.253–2.386)	0.660
Age	1.755 (0.606–5.084)	0.300
Grading	4.265 (0.5–36.345)	0.185
cT	1.014 (0.329–3.119)	0.981
cN	0.585 (0.205–1.671)	0.317
Microbial risk score	2.253 (1.483–3.422)	**<0.001**

The bold value indicates statistical significance.

## Discussion

Our study is the first to compare the intratumoral microbiome between pCR and non-pCR patients receiving nCRT. We observed significant differences in α and β diversities between the two groups. In addition, we identified the relative differences in the abundance of specific taxa between the pCR and non-pCR groups. The results revealed that the microbial profiles of non-pCR were characterized by higher α diversity. Further examination identified 12 significantly differential genera between the pCR and non-pCR groups: *Christensenella, Ruminococcus, Collinsella, Faecalibacterium, Akkermansia, Faecalitalea, Alistipes, Anaerostipes, Anaerobutyricum, Pavimonas, Blautia,* and *Bifidobacterium*. These 12 microbes were enriched in the non-pCR group, suggesting a negative correlation with the therapeutic response.

Based on RNA-seq data acquired from rectal cancer biopsy, our study first identified the presence of intratumoral microbiota in patients with LARC. In 2021, Liu et al. demonstrated an intratumoral microbial signature and revealed enriched microbiota, including Acidocacteria, Bacteroidetes, Cyanobacteria, Proteobacteria, Actinobacteria, Chloroflexi, Firmicutes, and Fusobacteria (Liu et al., 2021). Consistent with previous findings, the most abundant microbiomes detected at the phylum level in our work were Proteobacteria, Firmicutes, Bacteroidetes, Actinobacteria, and Fusobacteria. In addition, Liu et al. pointed out the heterogeneity between the intratumoral microbiome and the microbiome extracted from adjacent tissues, revealing distinct patterns between the intratumoral microbiome and gut microbiota. These results support the necessity of microbial identification from the tumor samples in our study and emphasize the importance of intratumoral microbiotas inside LARC. Previous studies focusing on the intratumoral microbes of LARC have revealed the role of microbiota in oncogenesis and tumor progression. However, limited investigations into the relationship between intratumoral microbiota and response to nCRT could not directly elucidate the causal clinical value of host-related microbes.

Since intratumoral microbiomes have potential differences from gut microbiomes, the predictive value of intratumoral microbiomes should be examined separately. Despite this, our intratumoral microbiota results were partially consistent with prior gut microbiome studies analyzing responses to nCRT. In a gut microbiome study (Yi et al., 2021), the family Coriobacteriaceae emerged as a crucial biomarker for non-responders. In our research, Coriobacteriaceae, composed predominantly of the genus *Collinesella*, likewise functioned as a biomarker for non-pCR. Another gut microbiome study noted the family Rikenellaceae and genus *Bacteroides* as the most abundant order in non-responders’ feces (Jang et al., 2020). Similarly, as the only genus from the family Rikenellacea uncovered within the intratumoral microbiome in our research, *Alistipes* had significant enrichment within the non-pCR group, and *Bacteroides* were also enriched in the non-pCR group but not significantly. Another study (Shi et al., 2020) reported the order Clostridiales, the genus *Faecalibacterium*, and the genus *Rumminococcus* as significantly enriched in non-responders, which is consistent with the results in our work. It’s important to highlight that among the 12 differential genera in our research, six belonged to the order Clostridiales.

Evidence has indicated that *Blautia* is more abundant in the shorter progression-free survival group of CRC (Martini et al., 2022). A study has also suggested that *Blautia* promotes the oncogenesis of LARC, possibly through its interaction with cholesteryl ester (Bisht et al., 2021). Similar to *Blautia*, *Parvimonas* has also been found to be involved in tumorigenesis (Liu et al., 2021). However, previous findings have suggested that *Faecalibacterium* (Dikeocha et al., 2022)*, Akkermansia* (Fan et al., 2021)*, Alistipes* (Dai et al., 2018)*, Anaerostipes* (Yuan et al., 2021), and *Bifidobacterium* (Lin et al., 2019) have anti-tumor patterns. These microbes are defined as beneficial commensal bacteria and are used as probiotic supplements in clinical practice. However, limited studies have revealed *Faecalibacterium* as a signature microbiota in CRC. In addition, network analysis revealed a positive relationship between anti-tumor microbes (including Firmicutes and Verrucomicrobia) and tumor-promoting microbes (Fusobacteria) in the non-pCR group (Figure 1F). These findings suggest that these “beneficial” microbes in the microbial dysbiosis mode of non-pCR patients could enhance the enrichment of tumor-promoting microbiotas and cause a general suppressor influence on the nCRT outcome. In conclusion, our study revealed that these ‘beneficial’ microbiotas might play an “evil role” for nCRT.

Combining multiple microbial signatures may increase the prognostic value of microbial profiles when considering the potential network among microbiomes. Thus, we defined a microbial risk score for LARC according to the abundance of the seven differential microbes at the genus level, namely *Collinsella*, *Alistipes*, *Christensenella*, *Faecalibacterium*, *Ruminococcus*, *Pavimonas*, and *Akkermansia*. The results revealed that the microbial risk score had an independent predictive value for nCRT response, indicating the clinical value of the intratumoral microbiome. To further understand the mechanism underlying the relationship between intratumoral microbiomes and the response to nCRT, we explored the correlation between microbiota and other essential factors in the TME, including tumor cells, infiltrating immune cells, and ECM. Previously, the correlation between CAFs and ECM was established in a study that observed the signature of CAF in the TME and defined the function of CAF based on gene expression and immunostaining (Lee et al., 2020). Moreover, ECM remodeling has also been linked to tumor treatment resistance in several studies (Drain et al., 2021; Pietilä et al., 2021; Fang et al., 2022). In our work, we explored five clusters of CAF subpopulations that presented potential interactions with important microbes, in which clusters 7 and 10 were found to be responsible for ECM formation, similar to another study reporting that microbiota inside CRC was co-localized with CAFs, activated the TLR2/NF-κB pathway in the CRC microenvironment (Xu et al., 2022). The above findings suggest a potential rationale that the intratumoral microbiota mediates the response to neoadjuvant therapy in LARC through interaction with CAFs, which play an essential role in ECM modulation.

It is essential to note some limitations of our study. Host genetic variation, ethnicity, geography, diet, lifestyle, and patterns of medications all play a role in the structure of the human microbiome (Yatsunenko et al., 2012; Goodrich et al., 2014; Brooks et al., 2018). Although our findings illustrated the microbial characteristics in the TME of LARC and the bacteria-associated signatures contributing to nCRT response prediction, the study is limited by its retrospective nature. It is currently constrained to a specific group of individuals from a developed country in Western Europe, given the currently limited RNA-seq data in patients with LARC who received nCRT. Besides, the predictive performance of the microbial risk score was not compared with that of the models or biomarkers proposed in previous studies (Kuo et al., 2012; Lim et al., 2015; Dayde et al., 2017) due to a lack of sufficient clinical and molecular data. Therefore, further research is warranted to verify the predictive value of the intratumoral microbiome of LARC for the responses to nCRT, especially in populations with different geological and ethnic backgrounds and complex medical histories. In addition, the analysis and collection of tumor samples in a time series would show the dynamic interaction between TME and intratumoral bacteria during the long process of nCRT. Future laboratory examinations could also focus on validating our conclusions on the relationship between the intratumoral microbiota and CAFs. Exploration of the complex crosstalk among intratumoral microbiota, tumor cells, and TME of patients with LARC may lead to the discovery of novel prognostic biomarkers, nutritious practices, and probiotic agents to improve the treatment effect for nCRT.

In conclusion, with considerable sample size, we performed systematic and comprehensive research on the intratumoral microbiome to predict the response to nCRT. The underlying mechanism of this finding could be related to the interactions between the intratumoral microbiota and CAFs. These results shed light on a new perspective of pre-treatment biomarkers for nCRT and could lead to better treatment strategies for LARC.

## Data availability statement

The datasets presented in this study can be found in online repositories. The names of the repository/repositories and accession number(s) can be found at: https://www.ncbi.nlm.nih.gov/, GSE190826; https://www.ncbi.nlm.nih.gov/, GSE132465.

## Ethics statement

Ethical approval was not required for the study involving humans in accordance with the local legislation and institutional requirements. Written informed consent to participate in this study was not required from the participants or the participants’ legal guardians/next of kin in accordance with the national legislation and the institutional requirements.

## Author contributions

YF and YS: study concept and design. LS, JQ, XK, YF, and YS: acquisition of data and obtained funding. LS, JQ, XK, and AG: analysis and interpretation of data. LS, JQ, XK, and AG: drafting of the manuscript. LS, JQ, XK, YuZ, HX, NL, JL, YaZ, AG, YF, and YS: critical revision of the manuscript for important intellectual content. LS, JQ, XK, HX, NL, and JL: statistical analysis. All authors contributed to the article and approved the submitted version.

## References

[ref1] Aklilu M. Eng C. (2011). The current landscape of locally advanced rectal cancer. Nat. Rev. Clin. Oncol. 8, 649–659. doi: 10.1038/nrclinonc.2011.118 21826084

[ref2] Bader G. D. Hogue C. W. (2003). An automated method for finding molecular complexes in large protein interaction networks. BMC Bioinform. 4:2. doi: 10.1186/1471-2105-4-2, PMID: 12525261 PMC149346

[ref3] Bisht V. Nash K. Xu Y. Agarwal P. Bosch S. Gkoutos G. V.. (2021). Integration of the microbiome, metabolome and transcriptomics data identified novel metabolic pathway regulation in colorectal Cancer. Int. J. Mol. Sci. 22:5763. doi: 10.3390/ijms22115763, PMID: 34071236 PMC8198673

[ref4] Bray F. Ferlay J. Soerjomataram I. Siegel R. L. Torre L. A. Jemal A. (2018). Global cancer statistics 2018: GLOBOCAN estimates of incidence and mortality worldwide for 36 cancers in 185 countries. CA Cancer J. Clin. 68, 394–424. doi: 10.3322/caac.21492, PMID: 30207593

[ref5] Brooks A. W. Priya S. Blekhman R. Bordenstein S. R. (2018). Gut microbiota diversity across ethnicities in the United States. PLoS Biol. 16:e2006842. doi: 10.1371/journal.pbio.2006842, PMID: 30513082 PMC6279019

[ref6] Buckley A. M. Lynam-Lennon N. O’Neill H. O’Sullivan J. (2020). Targeting hallmarks of cancer to enhance radiosensitivity in gastrointestinal cancers. Nat. Rev. Gastroenterol. Hepatol. 17, 298–313. doi: 10.1038/s41575-019-0247-2, PMID: 32005946

[ref7] Cercek A. Roxburgh C. S. D. Strombom P. Smith J. J. Temple L. K. F. Nash G. M.. (2018). Adoption of Total neoadjuvant therapy for locally advanced rectal Cancer. JAMA Oncol. 4:e180071. doi: 10.1001/jamaoncol.2018.0071, PMID: 29566109 PMC5885165

[ref8] Chen T. Liu Y. X. Huang L. (2022). ImageGP: An easy-to-use data visualization web server for scientific r esearchers. iMeta 1:e5. 10.1002/imt2.5 PMC10989750 38867732

[ref9] Da Cunha T. Vaziri H. Wu G. Y. (2022). Primary Sclerosing cholangitis and inflammatory bowel disease: a review. J. Clin. Transl. Hepatol. 10, 531–542. doi: 10.14218/JCTH.2021.00344, PMID: 35836773 PMC9240248

[ref10] Dai Z. Coker O. O. Nakatsu G. Wu W. K. K. Zhao L. Chen Z.. (2018). Multi-cohort analysis of colorectal cancer metagenome identified altered bacteria across populations and universal bacterial markers. Microbiome 6:70. doi: 10.1186/s40168-018-0451-2, PMID: 29642940 PMC5896039

[ref11] Davis N. M. Proctor D. M. Holmes S. P. Relman D. A. Callahan B. J. (2018). Simple statistical identification and removal of contaminant sequences in marker-gene and metagenomics data. Microbiome 6:226. doi: 10.1186/s40168-018-0605-2, PMID: 30558668 PMC6298009

[ref12] Dayde D. Tanaka I. Jain R. Tai M. C. Taguchi A. (2017). Predictive and prognostic molecular biomarkers for response to neoadjuvant Chemoradiation in rectal Cancer. Int. J. Mol. Sci. 18:573. doi: 10.3390/ijms18030573, PMID: 28272347 PMC5372589

[ref13] Dejea C. M. Fathi P. Craig J. M. Boleij A. Taddese R. Geis A. L.. (2018). Patients with familial adenomatous polyposis harbor colonic biofilms containing tumorigenic bacteria. Science 359, 592–597. doi: 10.1126/science.aah3648, PMID: 29420293 PMC5881113

[ref14] Dikeocha I. J. Al-Kabsi A. M. Chiu H. T. Alshawsh M. A. (2022). *Faecalibacterium prausnitzii* ameliorates colorectal tumorigenesis and suppresses proliferation of HCT116 colorectal Cancer cells. Biomedicine 10:1128. doi: 10.3390/biomedicines10051128, PMID: 35625865 PMC9138996

[ref15] Drain A. P. Zahir N. Northey J. J. Zhang H. Huang P. J. Maller O.. (2021). Matrix compliance permits NF-κB activation to drive therapy resistance in breast cancer. J. Exp. Med. 218:e20191360. doi: 10.1084/jem.20191360, PMID: 33822843 PMC8025243

[ref16] Fan L. Xu C. Ge Q. Lin Y. Wong C. C. Qi Y.. (2021). *A. muciniphila* suppresses colorectal tumorigenesis by inducing TLR2/NLRP3-mediated M1-like TAMs. Cancer Immunol. Res. 9, 1111–1124. doi: 10.1158/2326-6066.CIR-20-1019, PMID: 34389559

[ref17] Fang Y. Liang S. Gao J. Wang Z. Li C. Wang R.. (2022). Extracellular matrix stiffness mediates radiosensitivity in a 3D nasopharyngeal carcinoma model. Cancer Cell Int. 22:364. doi: 10.1186/s12935-022-02787-5, PMID: 36403050 PMC9675143

[ref18] Glynne-Jones R. Wyrwicz L. Tiret E. Brown G. Rödel C. Cervantes A.. (2017). Rectal cancer: ESMO clinical practice guidelines for diagnosis, treatment and follow-up. Ann. Oncol. 28, iv22–iv40. doi: 10.1093/annonc/mdx224, PMID: 28881920

[ref19] Goodrich J. K. Waters J. L. Poole A. C. Sutter J. L. Koren O. Blekhman R.. (2014). Human genetics shape the gut microbiome. Cells 159, 789–799. doi: 10.1016/j.cell.2014.09.053, PMID: 25417156 PMC4255478

[ref20] Han H. Cho J. W. Lee S. Yun A. Kim H. Bae D.. (2018). TRRUST v2: an expanded reference database of human and mouse transcriptional regulatory interactions. Nucleic Acids Res. 46, D380–D386. doi: 10.1093/nar/gkx1013, PMID: 29087512 PMC5753191

[ref21] Jang B. S. Chang J. H. Chie E. K. Kim K. Park J. W. Kim M. J.. (2020). Gut microbiome composition is associated with a pathologic response after preoperative chemoradiation in patients with rectal Cancer. Int. J. Radiat. Oncol. Biol. Phys. 107, 736–746. doi: 10.1016/j.ijrobp.2020.04.015, PMID: 32315676

[ref22] Kuo L. J. Chiou J. F. Tai C. J. Chang C. C. Kung C. H. Lin S. E.. (2012). Can we predict pathologic complete response before surgery for locally advanced rectal cancer treated with preoperative chemoradiation therapy? Int. J. Color. Dis. 27, 613–621. doi: 10.1007/s00384-011-1348-8 22080392

[ref23] Lee H. O. Hong Y. Etlioglu H. E. Cho Y. B. Pomella V. Van Den Bosch B.. (2020). Lineage-dependent gene expression programs influence the immune landscape of colorectal cancer. Nat. Genet. 52, 594–603. doi: 10.1038/s41588-020-0636-z, PMID: 32451460

[ref24] Li M. Xiao Q. Venkatachalam N. Hofheinz R. D. Veldwijk M. R. Herskind C.. (2022). Predicting response to neoadjuvant chemoradiotherapy in rectal cancer: from biomarkers to tumor models. Ther Adv Med Oncol 14:17588359221077972. doi: 10.1177/17588359221077972 35222695 PMC8864271

[ref25] Lim S. H. Chua W. Henderson C. Ng W. Shin J. S. Chantrill L.. (2015). Predictive and prognostic biomarkers for neoadjuvant chemoradiotherapy in locally advanced rectal cancer. Crit. Rev. Oncol. Hematol. 96, 67–80. doi: 10.1016/j.critrevonc.2015.05.003 26032919

[ref26] Lin P. Y. Li S. C. Lin H. P. Shih C. K. (2019). Germinated brown rice combined with *Lactobacillus acidophilus* and *Bifidobacterium animalis* subsp. *lactis* inhibits colorectal carcinogenesis in rats. Food Sci. Nutr. 7, 216–224. doi: 10.1002/fsn3.864, PMID: 30680175 PMC6341155

[ref27] Liu W. Zhang X. Xu H. Li S. Lau H. C. Chen Q.. (2021). Microbial community heterogeneity within colorectal neoplasia and its correlation with colorectal carcinogenesis. Gastroenterology 160, 2395–2408. doi: 10.1053/j.gastro.2021.02.020, PMID: 33581124

[ref28] Loos M. Quentmeier P. Schuster T. Nitsche U. Gertler R. Keerl A.. (2013). Effect of preoperative radio(chemo)therapy on long-term functional outcome in rectal cancer patients: a systematic review and meta-analysis. Ann. Surg. Oncol. 20, 1816–1828. doi: 10.1245/s10434-012-2827-z, PMID: 23269466

[ref29] Lu J. Breitwieser F. P. Thielen P. Salzberg S. L. (2017). Bracken: Estimating species abundance in metagenomics data. Peerj Comput. Sci. 2017:e104. doi: 10.7717/peerj-cs.104 PMC12016282 40271438

[ref30] Martini G. Ciardiello D. Dallio M. Famiglietti V. Esposito L. Corte C. M. D.. (2022). Gut microbiota correlates with antitumor activity in patients with mCRC and NSCLC treated with cetuximab plus avelumab. Int. J. Cancer 151, 473–480. doi: 10.1002/ijc.34033, PMID: 35429341 PMC9321613

[ref31] Nejman D. Livyatan I. Fuks G. Gavert N. Zwang Y. Geller L. T.. (2020). The human tumor microbiome is composed of tumor type-specific intracellular bacteria. Science 368, 973–980. doi: 10.1126/science.aay9189, PMID: 32467386 PMC7757858

[ref32] Newman A. M. Steen C. B. Liu C. L. Gentles A. J. Chaudhuri A. A. Scherer F.. (2019). Determining cell type abundance and expression from bulk tissues with digital cytometry. Nat. Biotechnol. 37, 773–782. doi: 10.1038/s41587-019-0114-2, PMID: 31061481 PMC6610714

[ref33] Nicolas A. M. Pesic M. Engel E. Ziegler P. K. Diefenhardt M. Kennel K. B.. (2022). Inflammatory fibroblasts mediate resistance to neoadjuvant therapy in rectal cancer. Cancer Cell 40, 168–184.e13. doi: 10.1016/j.ccell.2022.01.004, PMID: 35120600

[ref34] Parks D. H. Tyson G. W. Hugenholtz P. Beiko R. G. (2014). STAMP: statistical analysis of taxonomic and functional profiles. Bioinformatics 30, 3123–3124. doi: 10.1093/bioinformatics/btu494, PMID: 25061070 PMC4609014

[ref35] Petrelli F. Trevisan F. Cabiddu M. Sgroi G. Bruschieri L. Rausa E.. (2020). Total neoadjuvant therapy in rectal Cancer: a systematic review and Meta-analysis of treatment outcomes. Ann. Surg. 271, 440–448. doi: 10.1097/SLA.0000000000003471 31318794

[ref36] Pietilä E. A. Gonzalez-Molina J. Moyano-Galceran L. Jamalzadeh S. Zhang K. Lehtinen L.. (2021). Co-evolution of matrisome and adaptive adhesion dynamics drives ovarian cancer chemoresistance. Nat. Commun. 12:3904. doi: 10.1038/s41467-021-24009-8, PMID: 34162871 PMC8222388

[ref37] Poore G. D. Kopylova E. Zhu Q. Carpenter C. Fraraccio S. Wandro S.. (2020). Microbiome analyses of blood and tissues suggest cancer diagnostic approach. Nature 579, 567–574. doi: 10.1038/s41586-020-2095-1, PMID: 32214244 PMC7500457

[ref38] Pucciarelli S. Del Bianco P. Efficace F. Serpentini S. Capirci C. De Paoli A.. (2011). Patient-reported outcomes after neoadjuvant chemoradiotherapy for rectal cancer: a multicenter prospective observational study. Ann. Surg. 253, 71–77. doi: 10.1097/SLA.0b013e3181fcb856 21135694

[ref39] Rubinstein M. R. Baik J. E. Lagana S. M. Han R. P. Raab W. J. Sahoo D.. (2019). *Fusobacterium nucleatum* promotes colorectal cancer by inducing Wnt/β-catenin modulator Annexin A1. EMBO Rep. 20:e47638. doi: 10.15252/embr.201847638, PMID: 30833345 PMC6446206

[ref40] Sauer R. Becker H. Hohenberger W. Rödel C. Wittekind C. Fietkau R.. (2004). Preoperative versus postoperative chemoradiotherapy for rectal cancer. N. Engl. J. Med. 351, 1731–1740. doi: 10.1056/NEJMoa040694 15496622

[ref41] Segata N. Izard J. Waldron L. Gevers D. Miropolsky L. Garrett W. S.. (2011). Metagenomic biomarker discovery and explanation. Genome Biol. 12:R60. doi: 10.1186/gb-2011-12-6-r60, PMID: 21702898 PMC3218848

[ref42] Shi W. Shen L. Zou W. Wang J. Yang J. Wang Y.. (2020). The gut microbiome is associated with therapeutic responses and toxicities of neoadjuvant Chemoradiotherapy in rectal cancer patients-a pilot study. Front. Cell. Infect. Microbiol. 10:562463. doi: 10.3389/fcimb.2020.562463, PMID: 33363048 PMC7756020

[ref43] Traag V. A. Waltman L. Van Eck N. J. (2019). From Louvain to Leiden: guaranteeing well-connected communities. Sci. Rep. 9:5233. doi: 10.1038/s41598-019-41695-z, PMID: 30914743 PMC6435756

[ref44] Van Der Sluis F. J. Couwenberg A. M. De Bock G. H. Intven M. P. W. Reerink O. Van Leeuwen B. L.. (2020). Population-based study of morbidity risk associated with pathological complete response after chemoradiotherapy for rectal cancer. Br. J. Surg. 107, 131–139. doi: 10.1002/bjs.11324, PMID: 31625143

[ref45] Wang Y. Yang H. Xu H. (2022). Age-specific microbiota in altering host inflammatory and metabolic signaling and metabolome based on sex. Hepatobiliary Surg Nutr 11, 305–307. doi: 10.21037/hbsn-2022-04, PMID: 35464278 PMC9023829

[ref46] Wen T. Xie P. Yang S. Niu G. Liu X. Ding Z.. (2022). ggClusterNet: An R package for microbiome network analysis and modularity-based multiple network layouts. iMeta 1:e32. doi: 10.1002/imt2.32 PMC10989811 38868720

[ref47] West M. A. Loughney L. Barben C. P. Sripadam R. Kemp G. J. Grocott M. P.. (2014). The effects of neoadjuvant chemoradiotherapy on physical fitness and morbidity in rectal cancer surgery patients. Eur. J. Surg. Oncol. 40, 1421–1428. doi: 10.1016/j.ejso.2014.03.021, PMID: 24784775

[ref48] Wood D. E. Lu J. Langmead B. (2019). Improved metagenomic analysis with kraken 2. Genome Biol. 20:257. doi: 10.1186/s13059-019-1891-0, PMID: 31779668 PMC6883579

[ref49] Xu Z. Lv Z. Chen F. Zhang Y. Xu Z. Huo J.. (2022). Dysbiosis of human tumor microbiome and aberrant residence of Actinomyces in tumor-associated fibroblasts in young-onset colorectal cancer. Front. Immunol. 13:1008975. doi: 10.3389/fimmu.2022.1008975, PMID: 36119074 PMC9481283

[ref50] Yatsunenko T. Rey F. E. Manary M. J. Trehan I. Dominguez-Bello M. G. Contreras M.. (2012). Human gut microbiome viewed across age and geography. Nature 486, 222–227. doi: 10.1038/nature11053, PMID: 22699611 PMC3376388

[ref51] Yi Y. Shen L. Shi W. Xia F. Zhang H. Wang Y.. (2021). Gut microbiome components predict response to neoadjuvant chemoradiotherapy in patients with locally advanced rectal cancer: a prospective, longitudinal study. Clin. Cancer Res. 27, 1329–1340. doi: 10.1158/1078-0432.CCR-20-3445, PMID: 33298472

[ref52] Yuan X. Xue J. Tan Y. Yang Q. Qin Z. Bao X.. (2021). Albuca Bracteate polysaccharides synergistically enhance the anti-tumor efficacy of 5-fluorouracil against colorectal Cancer by modulating β-catenin signaling and intestinal Flora. Front. Pharmacol. 12:736627. doi: 10.3389/fphar.2021.736627, PMID: 34552494 PMC8450769

[ref53] Zhang M. Lv Y. Hou S. Liu Y. Wang Y. Wan X. (2021). Differential mucosal microbiome profiles across stages of human colorectal cancer. Life (Basel) 11:831. doi: 10.3390/life11080831, PMID: 34440574 PMC8401903

