# Supplementary material for: Interaction between intratumoral microbiota and tumor mediates the response of neoadjuvant therapy for rectal cancer
Source: Front Microbiol. 2023 Oct 12;14:1229888. doi: 10.3389/fmicb.2023.1229888 (PMC10602640; doi:10.3389/fmicb.2023.1229888)
Supplement: Supplementary file 1 [file Data_Sheet_1.docx]

**Supplementary Materials**

**Supplementary Table 1.** The predictive value of 12 differential genera for patients’ responses to neoadjuvant chemoradiation therapy (related to Figure 5A).

| **Genera** | **AUC** | **95% CI** | ***p*-value** |
| --- | --- | --- | --- |
| *Bifidobacterium* | 0.624 | 0.505–0.744 | 0.058 |
| *Collinsella* | 0.629 | 0.521–0.737 | 0.049 * |
| *Alistipes* | 0.685 | 0.580–0.789 | 0.005 * |
| *Christensenella* | 0.672 | 0.570–0.773 | 0.009 * |
| *Anaerobutyricum* | 0.620 | 0.503–0.738 | 0.067 |
| *Anaerostipes* | 0.589 | 0.474–0.704 | 0.174 |
| *Blautia* | 0.623 | 0.506–0.739 | 0.061 |
| *Faecalibacterium* | 0.647 | 0.541–0.752 | 0.025 * |
| *Ruminococcus* | 0.645 | 0.535–0.755 | 0.027 * |
| *Faecalitalea* | 0.614 | 0.503–0.726 | 0.081 |
| *Parvimonas* | 0.682 | 0.577–0.787 | 0.005 * |
| *Akkermansia* | 0.693 | 0.586–0.799 | 0.003 * |

Asterisks indicate statistical significance. Aberrations: AUC, area under receiver operating characteristics curve; CI, confidential interval.

**Supplementary Table 2.** The predictive value of seven differential genera after patients being stratified by the corresponding cutoff values based on the relative abundance of these microbes (related to Supplementary Figure 4).

| **Genera** | **AUC** | **95% CI** | ***p*-value** |
| --- | --- | --- | --- |
| *Collinsella* | 0.652 | 0.545–0.759 | 0.021 * |
| *Alistipes* | 0.702 | 0.601–0.804 | 0.002 * |
| *Christensenella* | 0.702 | 0.595–0.809 | 0.002 * |
| *Faecalibacterium* | 0.658 | 0.548–0.768 | 0.016 * |
| *Ruminococcus* | 0.652 | 0.543–0.761 | 0.021 * |
| *Parvimonas* | 0.669 | 0.549–0.789 | 0.010 * |
| *Akkermansia* | 0.689 | 0.576–0.801 | 0.004 * |

Asterisks indicate statistical significance. Aberrations: AUC, area under receiver operating characteristics curve; CI, confidential interval.


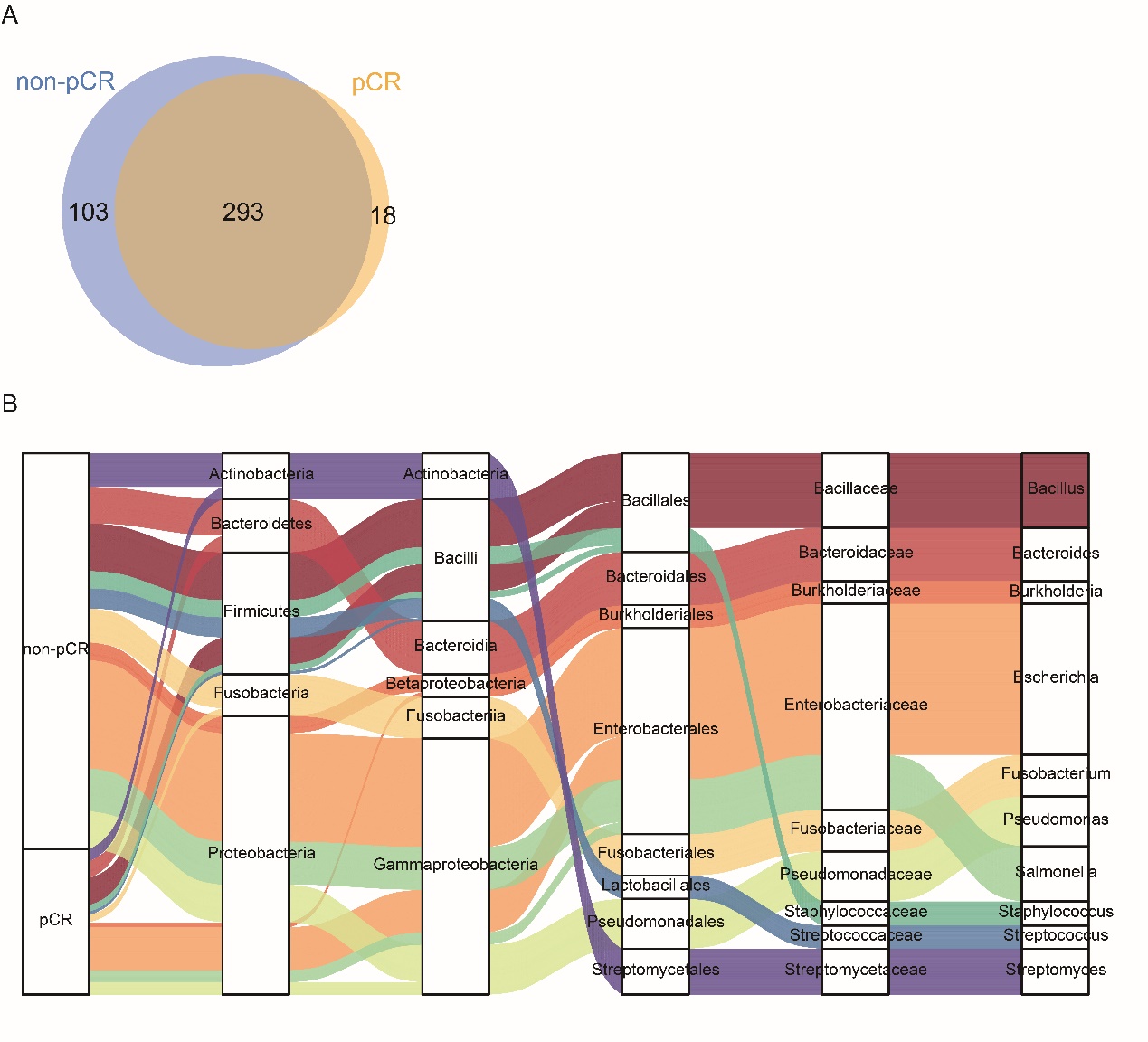


**Supplementary Figure 1.** (A) Venn map showing the number of genera in each group. (B) Sankey map showing the taxonomic information of the top ten genera at different levels.


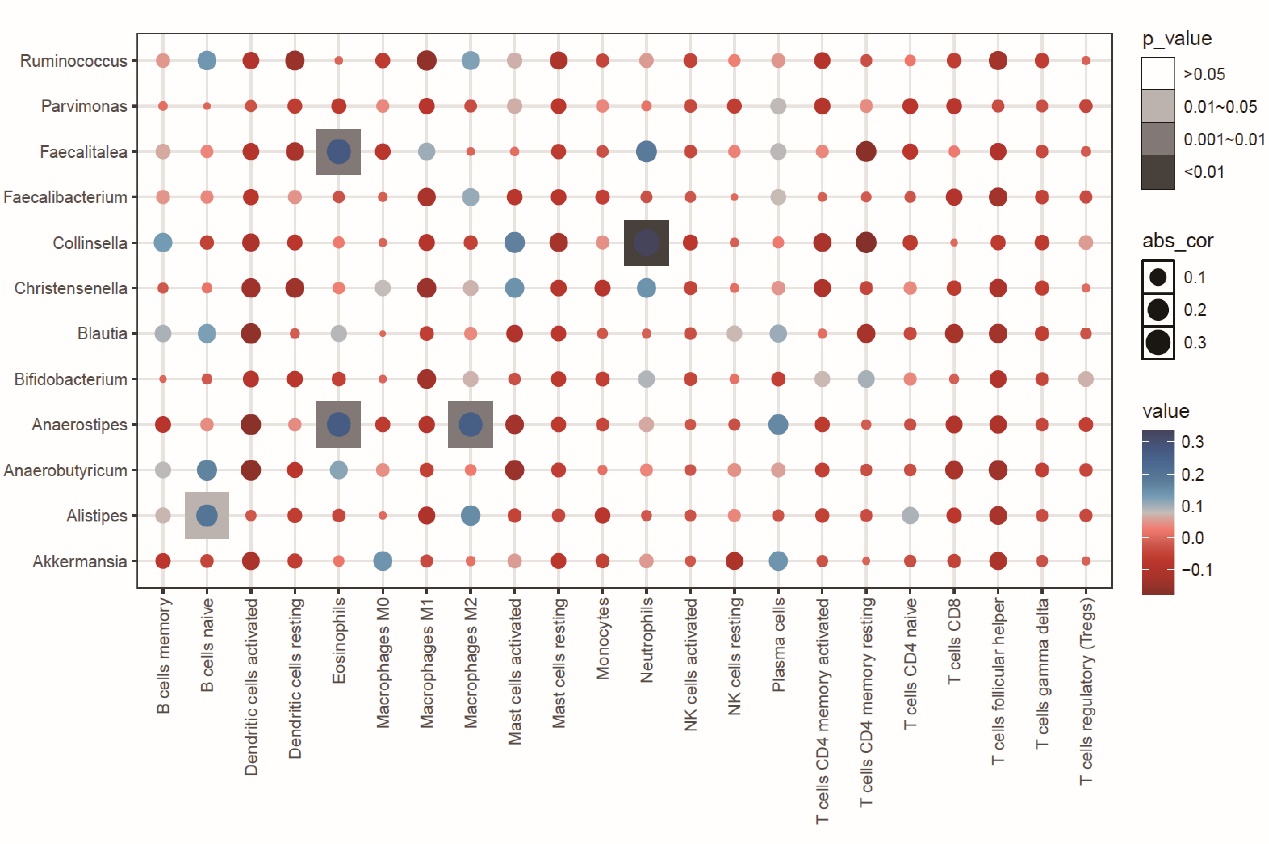


**Supplementary Figure 2.** Heatmap indicates the correlation between immune cell infiltration and the 12 differential microbes.


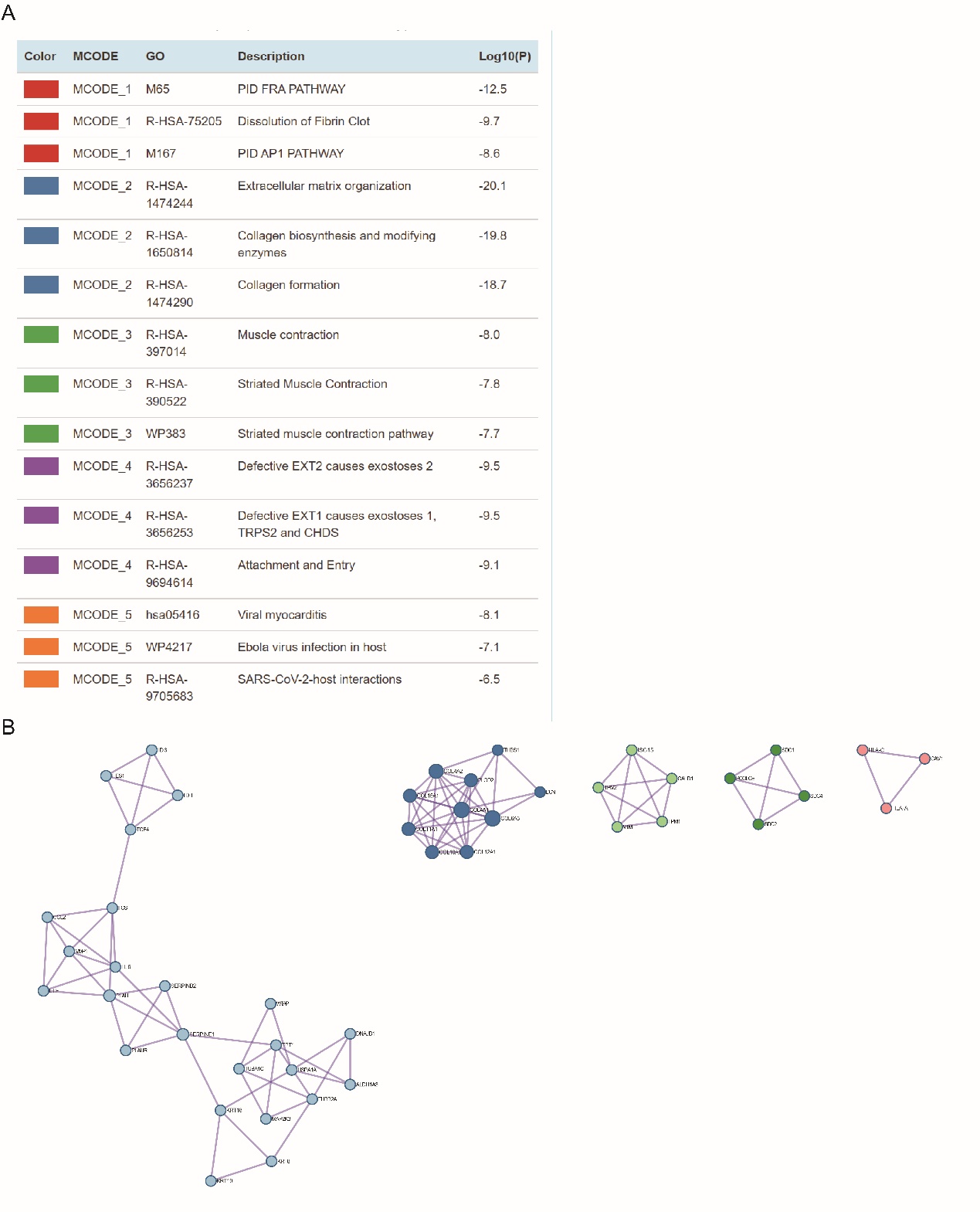


**Supplementary Figure 3.** (A) GO enrichment analysis of MCODE network components of the marker genes of CAF cluster 10. (B) Protein-protein physical interactions among the signature genes of CAF cluster 10.


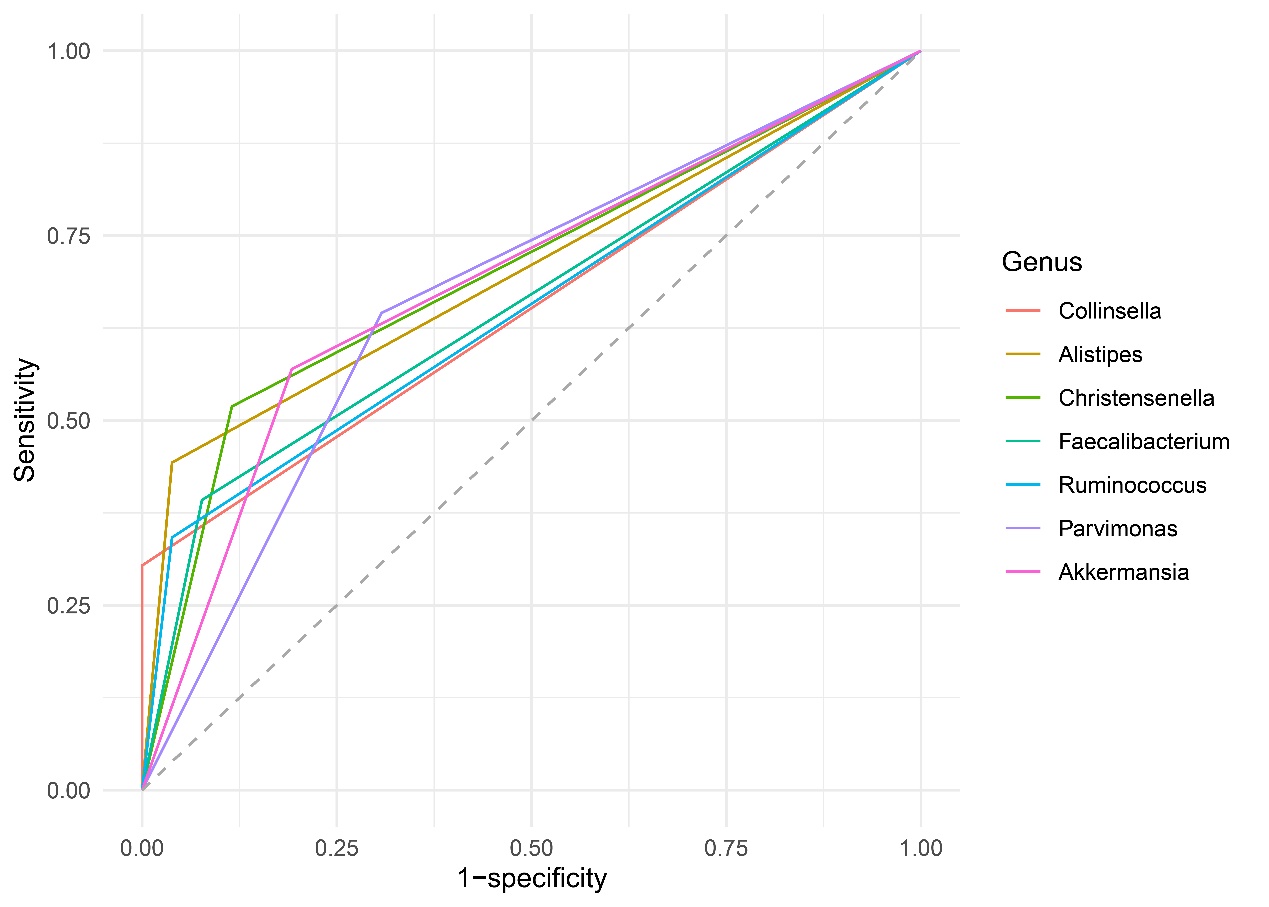


**Supplementary Figure 4.** Receiver operating characteristics analysis of seven differential microbes for predicting the nCRT responses after patients are stratified by the corresponding cutoff values based on the relative abundance of these microbes (related to Supplementary Table 2).
